# Molecular Regulation and Oncogenic Functions of TSPAN8

**DOI:** 10.3390/cells13020193

**Published:** 2024-01-19

**Authors:** Jicheng Yang, Ziyan Zhang, Joanne Shi Woon Lam, Hao Fan, Nai Yang Fu

**Affiliations:** 1 Cancer and Stem Cell Biology Program, Duke-NUS Medical School, Singapore 169857, Singapore; 2 ACRF Cancer Biology and Stem Cells Division, The Walter and Eliza Hall Institute of Medical Research, Parkville, VIC 3052, Australia; 3 Department of Medicine, University of Melbourne, Parkville, VIC 3010, Australia; 4 Bioinformatics Institute (BII), Agency for Science, Technology and Research (A*STAR), Singapore 138671, Singapore; 5 Department of Physiology, National University of Singapore, Singapore 117593, Singapore

**Keywords:** TSPAN8, tetraspanin, tetraspanin-enriched microdomain, plasma membrane, cell signaling, exosome, stem cell, cancer stem cell, tumor-associated antigen, cell invasion, cancer metastasis

## Abstract

Tetraspanins, a superfamily of small integral membrane proteins, are characterized by four transmembrane domains and conserved protein motifs that are configured into a unique molecular topology and structure in the plasma membrane. They act as key organizers of the plasma membrane, orchestrating the formation of specialized microdomains called “tetraspanin-enriched microdomains (TEMs)” or “tetraspanin nanodomains” that are essential for mediating diverse biological processes. TSPAN8 is one of the earliest identified tetraspanin members. It is known to interact with a wide range of molecular partners in different cellular contexts and regulate diverse molecular and cellular events at the plasma membrane, including cell adhesion, migration, invasion, signal transduction, and exosome biogenesis. The functions of cell-surface TSPAN8 are governed by ER targeting, modifications at the Golgi apparatus and dynamic trafficking. Intriguingly, limited evidence shows that TSPAN8 can translocate to the nucleus to act as a transcriptional regulator. The transcription of *TSPAN8* is tightly regulated and restricted to defined cell lineages, where it can serve as a molecular marker of stem/progenitor cells in certain normal tissues as well as tumors. Importantly, the oncogenic roles of TSPAN8 in tumor development and cancer metastasis have gained prominence in recent decades. Here, we comprehensively review the current knowledge on the molecular characteristics and regulatory mechanisms defining TSPAN8 functions, and discuss the potential and significance of TSPAN8 as a biomarker and therapeutic target across various epithelial cancers.

## 1. Introduction

The tetraspanin superfamily of proteins is characterized by four intramembrane domains, two extracellular loops (one large and one small), one small inner loop, and short N- and C-terminal intracellular tails [1]. To date, a total of 33 members have been identified and characterized in the human tetraspanin superfamily. These proteins have been designated as TSPAN1–33, although researchers continue to use the original names for certain members, such as CD9, CD63, CD81, CD82 and CD151 [2]. Some tetraspanins, including CD63, CD81 and CD151, are present in nearly all cell and tissue types across diverse animal species. However, the expression of other tetraspanins appears to be restricted to specific tissues or cell types [3]. Over the last three decades, tetraspanins have been shown to play a role in a multitude of biological processes, such as fertilization, parasite and viral infection, mental health, immune response, and tumor development [1,4]. Dysfunctions or abnormities have been noted to be the result of genetic modifications of certain tetraspanin members in mouse, fly, worm and fungi [4]. Furthermore, mutations in at least four tetraspanin members, TSPAN7, TSPAN22, TSPAN23 and CD151, have been linked to different human diseases, such as mental retardation syndromes [5,6], retinitis pigmentosa [7,8,9,10], and hereditary nephritis [11].

Initially identified in mammals during the search for novel cell-surface antigens associated with human cancer cells, tetraspanins are evolutionarily conserved proteins. This review will focus on TSPAN8, which is one of the earliest tetraspanin members to be identified. A plasma membrane protein, targeted by a monoclonal antibody (CO-029) derived from mice immunized with human gastrointestinal tumor cells, was identified in 1990 [12]. Initially named CO-029, the protein was subsequently referred to as transmembrane 4 superfamily 3 (TM4SF3), as it contains four transmembrane domains—a common structural feature of the integral membrane protein family [13]. A rat protein called D6.1A—identified in 1998 using the D6.1 monoclonal antibody from a group of antigens specifically expressed in metastasizing tumor cells in rat cancer models—displayed 70% similarity to CO-029 at both the DNA and protein levels [14]. These homologs are now commonly designated as TSPAN8, although they are occasionally referred to by their earlier names (CO-029/TM4SF3/D6.1A), even in some recent papers. Studies over the past 30 years have provided critical insights into the putative plasma membrane topology, expression patterns, molecular regulation, post-translational modifications, binding partners, and the trafficking of TSPAN8. It is involved in a broad array of biological processes, including cell migration and invasion, signal transduction and exosome biogenesis. It acts as a key organizer of the plasma membrane, orchestrating the formation of the tetraspanin-enriched microdomains (TEMs). Surprisingly, recent studies have suggested that TSPAN8 can translocate from the plasma membrane to the nucleus, where it executes novel functions in transcriptional regulation. The diverse functions of TSPAN8 may contribute to its critical oncogenic roles in multiple stages of tumor development. Here, we review the current understanding of the molecular characteristics and regulatory mechanisms that underlie TSPAN8 functions. Building on the existing evidence, we also explore the potential of harnessing TSPAN8 as a molecular biomarker and therapeutic target for the diagnosis and treatment of various epithelial cancers.

## 2. TSPAN8 Is a Member of the Tetraspanin Superfamily

**Figure 1:**
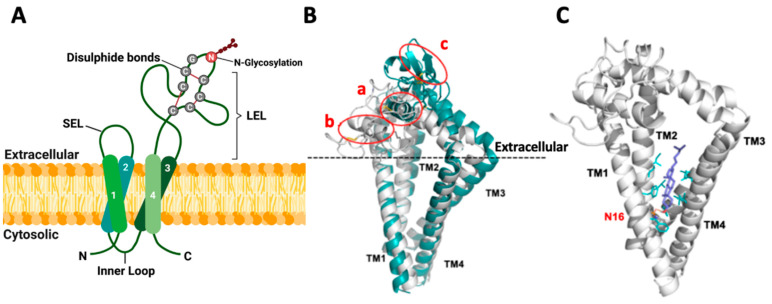
Topology and structure of TSPAN8 in the membrane. (**A**) Schematic diagram for the topology of human TSPAN8 in the plasma membrane. TSPAN8 contains four transmembrane domains (TM1-4), one inner loop and two extracellular loops (SEL: small extracellular loop and LEL: large extracellular loop). The six cysteine residues, with two in the conserved CCG motif of the tetraspanin superfamily, form three disulfide bonds as indicated. N118 in LEL is the conserved and sole N-glycosylation site of TSPAN8. Created with BioRender.com, accessed on 16 January 2024. (**B**) Overlap of TSPAN8 structures generated by AlphaFold (green) modeling and the TSPAN8 homology modeling (HM6K4J, white) based on CD9 template (PDB ID: 6K4J), with three disulfide bonds in the LEL domain for both. In HM6K4J, the LEL domain is more loosely packed and located nearer to the extracellular membrane plane than that of the AlphaFold model. The AlphaFold model forms a tightly packed LEL architecture, with a longer alpha helix formed between residues Glu133 to Phe149 (circled as a in figure) compared to the same region in HM6K4J, which is located closer to the extracellular membrane plane, and it has a shorter alpha helix formed between residues Glu133 to Ala141 instead (circled as b in figure). One β-sheet is formed within the region from residues Cys181 to Tyr190 in the AlphaFold model (circled as c) but not in HM6K4J. Furthermore, compared to HM6K4J, the TM2, TM3 and TM1 are further away from each other in the AlphaFold model. (**C**) Docking conformation of cholesterol in the TSPAN8 homology model HM6K4J. In contrast to crystal structure of CD81 (PDB ID: 5TCX), the β-hydroxyl group of the docked cholesterol in HM6K4J interacts with Asn16 (N16) sidechain (orange) through H-bonding, probably because Glu219 in CD81 is not conserved in TSPAN8 but replaced by Gly223. This may lead to the β-face of cholesterol turning towards TM1 and TM2 instead. The rigid sterol ring of cholesterol is also further stabilized by hydrophobic sidechains (cyan sticks) of residues at the binding site: Phe15/Phe19/Phe88, Leu69/Leu92/Leu216 and Ile65/Ile66/Ile95/Ile219.

Despite the low overall protein sequence homology, tetraspanins exhibit a conserved structural topology in the plasma membrane (Figure 1A, with TSPAN8 as an example) that is primarily characterized by the presence of four transmembrane domains (TM1-4). However, this structural feature alone is not sufficient to classify an integral membrane protein as a tetraspanin superfamily member. Many other proteins outside the superfamily, such as CD20, sarcospan, connexins, claudins, TM4SF1, TM4SF4 and TM4SF5, also contain four transmembrane domains but lack other key characteristics of tetraspanins [13]. Of note, tetraspanins contain two well-defined and unique extracellular regions of varying sizes: a small extracellular loop (SEL) comprising 20 to 28 amino acids and a large extracellular loop (LEL) comprising 76 to 131 amino acids [15]. Molecular predictions based on sequence alignments and homology modeling [16,17,18] have revealed that certain polar amino acid residues within the TM domains, including Asn, Glu and Gln, are highly conserved and essential for stabilizing the topology and structure of tetraspanins in the plasma membrane. The extracellular domains are the most variable regions across tetraspanin members [19]. Intriguingly, the cysteine-cysteine-glycine (CCG) motif within the LEL domain is a highly conserved feature of tetraspanins. Moreover, tetraspanins can be further divided into different subclasses based on the number of cysteine residues (ranging from four to eight) in their LEL domains. TSPAN8, with six cysteine residues, belongs to the TspanC6 subclass that comprises as many as 20 of the total 33 human tetraspanin members [2]. Some studies suggest that different members within a tetraspanin subclass are functionally similar or redundant, as they interact with the same binding partners [20]. However, further evidence is required to confirm this hypothesis, and it remains highly possible that distinct members of tetraspanins, even within the same subclass, may have their own unique functions. In addition to the extracellular loops and transmembrane domains, tetraspanins also contain three cytoplasmic segments, including a small intracellular loop that connects TM2 and TM3 and short N-terminal and C-terminal tails [1]. However, the roles of these cytoplasmic regions in defining the functions of different tetraspanins in the plasma membrane remain largely unclear.

## 3. Topology and Structure of TSPAN8 in the Plasma Membrane

Crystallographic studies have resolved the topology and structure of human full-length CD81 (PDB ID: 5TCX), CD9 (PDB ID: 6K4J) and CD53 (PDB ID: 6WVG), three tetraspanin members belonging to the TspanC4 subclass [21,22,23]. The four TM domains in these proteins form a reversed cone-like molecular shape, which is likely the canonical TM domain architecture shared by all tetraspanins. This unique structure may contribute to the generation of the curvature and remodeling of the plasma membrane. Moreover, the two pairs of helices formed by TM1/TM2 and TM3/TM4, respectively, create an intramembrane cavity for binding a lipid molecule such as cholesterol and monoolein, and the physical interactions with lipid molecules likely regulate tetraspanin functions. The LEL domains of CD81, CD9 and CD53 exhibit a similar overall mushroom-like structure composed of five helices, termed A to E. The A and E helices make up the “stalk” of the mushroom, while the helix B and the variable C–D region form its “head”. Interestingly, the LEL domain of CD81 alone has a similar structure to that of the LEL in the full-length CD81 protein [18], suggesting that the TM domains are not critical for LEL conformation. The five LEL helices are stabilized by the formation of two conserved intramolecular disulfide bonds between the four cysteine residues in these three tetraspanin members, pointing to a critical role for the conserved cysteines in shaping the secondary and tertiary structures of the LEL domain of tetraspanins [4]. Notably, some monoclonal antibodies against the LEL domains of tetraspanins can only recognize them under non-reducing conditions in Western blotting analysis, which clearly indicates the formation of intramolecular disulfide bonds by cysteines within LEL domains under physiological conditions. In line with crystallographic analysis, a study employing multiple sequence alignments and homology modeling approaches also predicted a similar structure for the LEL domains of distinct tetraspanin members and revealed that the LEL domain comprises two subdomains: one subdomain (helix A, B and E) exhibiting a structurally conserved fold and the other (helix C and D) displaying significant variability among different tetraspanin proteins that result in diversity in their interacting partners and functions [17,24]. Moreover, molecular dynamics simulation analyses of full-length tetraspanins suggest that the LEL domain displays a large rotation capacity and can adopt two different conformations: open and closed. In the open conformation, the LEL domain orientates and interacts with the SEL domain, which makes the protein more exposed and accessible for interactions with other protein partners. In contrast, the LEL domain in the closed conformation disengages from the SEL domain and rotates toward the membrane, a mechanism that prevents its interaction with protein partners. Intriguingly, the native CD53 protein adopts an open conformation due to the constitutive interaction between the SEL and LEL domains. Furthermore, the binding of cholesterol is critical for maintaining the open structure of CD81 but does not affect the rotation of the LEL domain of CD53. Overall, further research is needed to investigate the specific conditions or regulatory mechanisms that determine tetraspanin conformations.

To get some insights into the potential topology and structure of human TSPAN8, we conducted both homology (based on the CD9/6K4J template) and AlphaFold modeling (Figure 1B). Three disulfide bonds (Cys152/Cys172, Cys151/Cys194 and Cys174/Cys181) in the LEL domain were generated in both models. TM conformation and the overall structures derived from these two modeling methods were similar, displaying many typical structural features of tetraspanins. Ligand docking analysis performed on the homology model also suggested that cholesterol is effectively accommodated within the transmembrane domains of TSPAN8 (Figure 1C). However, the two models presented several differences in the LEL domain structures, and further studies are needed to determine whether one of the models represents TSPAN8 structure more accurately or whether they represent different conformations.

## 4. Regulation of ER Targeting, N-Glycosylation and Cell-Surface Expression of TSPAN8

In mammalian cells, nascent polypeptides are targeted to the endoplasmic reticulum (ER) membrane through a co-translational or post-translational mechanism. In the co-translational system, an SRP (signal recognition particle) receptor recognizes the N-terminal signal peptide of a precursor polypeptide, upon which ribosomes are guided to the ER membrane to complete the translation process [25]. In contrast, post-translational targeting refers to the process that occurs after a precursor polypeptide has been fully synthesized and released from the ribosome. It involves either a C-terminal signal, known as the transmembrane helix (TMH), or the N-terminal signal peptide, and it is mediated by GET (guided entry of tail-anchored) proteins. In addition to the SRP and GET routes, recent studies revealed a new SRP-independent (SND) ER targeting pathway that can regulate both co-translational and post-translational ER targeting [26]. Despite the existence of multiple pathways, the mechanism governing the ER targeting of tetraspanins is poorly understood, as they do not possess a signal peptide sequence at their N-terminus or TMH at their C-terminus for ER targeting. We recently identified TMEM208 as a regulator of the cell-surface presentation of TSPAN8 and several other tetraspanins [27]. TMEM208 is the human orthologue of Snd2 [28], which, together with Snd1 and Snd3, are key proteins regulating the SND ER-targeting pathway in yeast [26]. However, further investigation is needed to determine whether TSPAN8 and other tetraspanins are targeted to the ER through SND. Interestingly, deleting TM domains in different tetraspanins led to their retention within the ER, implying a pivotal role for the TM regions in facilitating the exit of tetraspanin from the ER [29,30,31].

Most of the plasma membrane proteins are glycosylated in eukaryotes through N-glycosylation, which is a complex biosynthesis pathway that attaches oligosaccharides to the amide nitrogen of asparagine (Asn) residue in proteins. It is initiated at the ER and maturated at the Golgi apparatus through a cascade of enzymatic processes: a lipid-linked oligosaccharide generated by multiple enzymes is transferred onto the N-glycosylation site of a nascent polypeptide by oligosaccharyltransferases at the ER. Glucose residues on the branch terminals are subsequently removed by glucosidases. Further removal of a mannose residue by α-mannosidase permits the export of high mannose glycoproteins to the Golgi apparatus for glycan branch maturation. This process is facilitated by a sequential cascade of glycosidases and glycosyltransferases within the Golgi, which ultimately shape and determine the glycan profile of N-glycoproteins [32]. The composition of glycans in mature glycoproteins is highly heterogeneous and is dictated by the availability of numerous glycosidases and glycosyltransferases during the transit of proteins through the ER and Golgi. N-glycosylation is implicated in various biological processes, such as the folding, cell-surface expression, secretion and transport of proteins, receptor interaction and endocytosis. It is known to occur at the consensus Asn-X-Ser/Thr sequence, where X can be any residue except for proline. Most of the 33 human tetraspanin members, except for TSPAN12/16/20/23/28/32, are predicted to contain at least one conserved N-glycosylation site at their SEL or LEL domains. Notably, CD9 is the only tetraspanin that harbors a predicted N-glycosylation site in the SEL, but not in the LEL domain [33]. However, our recent work did not detect CD9 N-glycosylation in the tested cells, suggesting that the prediction is false and that CD9 is not modified by N-glycosylation [27].

To unravel the regulatory mechanisms governing the cell-surface expression of tetraspanins in human cells in an unbiased manner, we recently conducted genome-wide loss-of-function CRISPR-Cas9 screens, using the cell-surface expression levels of TSPAN8 as a readout. Our screens identified N-glycosylation as an important mechanism in controlling the cell-surface presentation of TSPAN8 [27]. The N118 residue in the human TSPAN8 protein was further identified as the sole N-glycosylation site that is evolutionarily conserved across various species [27]. Unexpectedly, the presence of biantennary N-glycans, but not more complex glycan structures or core fucosylation, was found to be important for the cell-surface expression of TSPAN8 and other tetraspanins in the tested cell lines. Interestingly, TSPAN8 was rapidly internalized when the N-glycosylation process was blocked.

Further to N-glycosylation, tetraspanin proteins are known to undergo palmitoylation, a process by which fatty acids are covalently attached to cysteine (less frequently to serine and threonine) residues of membrane proteins in the Golgi. The membrane-proximal cysteines have been recognized as conserved sites across tetraspanins. Biochemical analysis suggests that the palmitoylation of tetraspanins facilitates their interactions with membrane receptors, which leads to the assembly of TEMs [34,35]. However, super-resolution microscopy data suggest that nanoscale clustering of CD9 on the cell surface is not affected by lipidation [36]. Moreover, blocking palmitoylation in a TSPAN8 mutant where all the juxtamembrane cysteine residues were replaced by alanine (Tspan8-Δpalmitoylation) did not affect the TSPAN8 presentation in the plasma membrane [37]. In line with this, the elimination of palmitoylation sites in other tetraspanins did not significantly impair their trafficking to the plasma membrane [34,36,38]. Moreover, our CRISPR-Cas9 screens did not identify any enzyme critical for palmitoylation as a regulator for the cell-surface presentation of tetraspanins. Nevertheless, a recent study suggests that palmitoylation accelerates the extraction of TSPAN8 from the plasma membrane [39].

## 5. Tetraspanin-Enriched Microdomains Regulated by TSPAN8 in the Plasma Membrane

A large body of evidence suggests that the diverse biological functions of tetraspanins are due to their ability to interact with a wide array of partners, forming distinct molecular complexes in the plasma membrane. Co-immunoprecipitation studies, using mild detergents, have revealed the formation of protein complexes between tetraspanins and various protein partners [40,41]. Microscopy techniques such as fluorescence resonance energy transfer (FRET) have further confirmed the presence of these complexes on the plasma membrane [42]. These primary complexes are known to engage in a network of secondary interactions to form a tetraspanin ‘web’, which have also been referred to as “tetraspanin-enriched microdomains” (TEMs) [43]. They have recently been named “tetraspanin nanodomains” following super-resolution microscopy studies that revealed their diameters to be in the nanometer range (70–150 nm) [44]. Within the core of these specialized microdomains, multiple tetraspanins interact with each other and other membrane proteins, forming networks with context-dependent roles. The precise composition of TEMs is not yet fully elucidated and is likely very dynamic.

**Figure 2:**
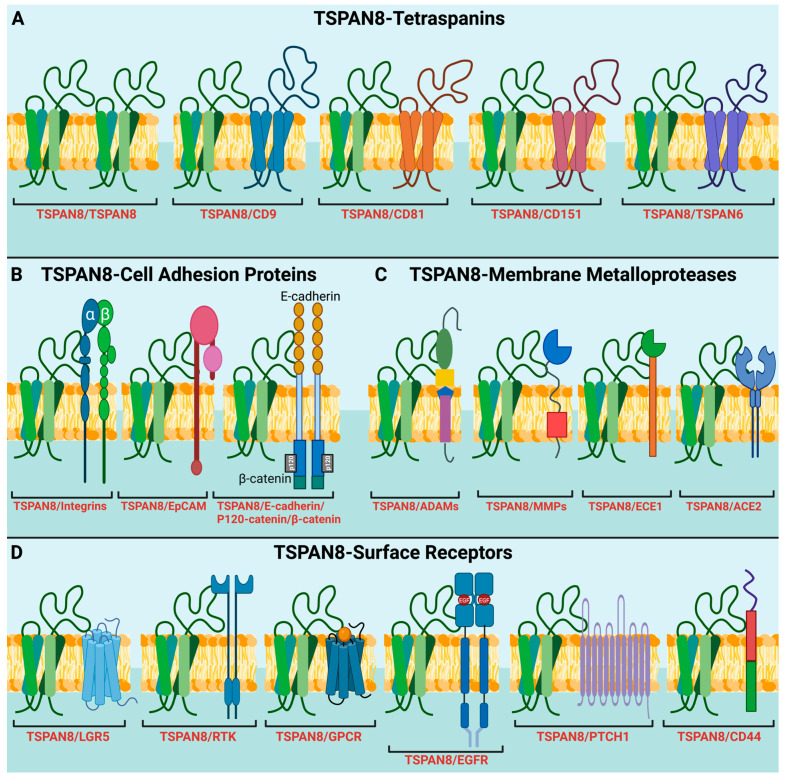
TSPAN8 recruits different protein partners into TEMs for diverse molecular and signaling processes. (**A**) Interaction of TSPAN8 with different tetraspanin members. (**B**) Interaction of TSPAN8 with adhesion proteins, including β1 integrins, EpCAM, E-cadherin, p120-catenin, and β-catenin. These interactions are involved in regulating cell adhesion, migration, and cell signaling, potentially contributing to tumor progression and metastasis. (**C**) Interaction of TSPAN8 with distinct membrane metalloproteases, such as ADAMs, MMPs, ECE1 and ACE2. (**D**) Interaction of TSPAN8 with different cell-surface receptors, participating in receptor-mediated signaling pathways. These receptors include LGR5, RTKs (receptor tyrosine kinases), GPCRs (G-protein coupled receptors), EGFR, PTCH1 (protein patched homolog 1) and CD44. Of note is that all the interactions shown here are based on published papers. Some interactions may be indirect. Created with BioRender.com, accessed on 19 January 2024.

The interactions between tetraspanins can be homophilic, such as TSPAN8-TSPAN8, or heterophilic, such as TSPAN8-CD151 (Figure 2A). Using monoclonal antibodies specifically against the CD9 in human cells, tetraspanin complexes were isolated via immunoaffinity purification and analyzed using mass spectrometry. The study identified 32 CD9-interacting proteins, including TSPAN8 and six other tetraspanins (CD81, CD151 and TSPAN1/9/14/15) [45]. A similar study in rat carcinoma confirmed TSPAN8 interactions with CD9, CD81 and CD151, as well as revealed non-tetraspanin partners, including α3β1 integrins and other signaling molecules, such as the Ig superfamily member FPRP (prostaglandin F2α receptor regulatory protein), type II PI4K (phosphatidylinositol 4-kinase) and EpCAM (epithelial cell adhesion molecule) [46]. More recently, around 60 membrane proteins, including several tetraspanins, were pulled down by an anti-TSPAN8 antibody and were identified as TSPAN8 binding partners in a human colon cancer cell line using a similar methodology [47]. The interactions of TSPAN8 with other molecular partners and their involvement in different molecular and cellular processes will be discussed in more detail in the latter sections of this review. Notably, super-resolution microscopy-based studies revealed that nanoclusters are formed by up to 10 molecules of a single tetraspanin member, such as CD53, CD81 and CD82, rather than multiple different members [48]. However, TSPAN8 was shown to exist in a monomer–dimer equilibrium within the plasma membrane of living cells [37], where dimerization is characterized by a high dissociation constant. This suggests a low binding affinity between TSPAN8 molecules and that TSPAN8 may preferentially interact with other partners within the TEMs. However, it remains unclear how the monomer–dimer equilibrium of TSPAN8 within the plasma membrane influences its interactions with other molecules within the TEMs.

In addition to protein partners, tetraspanins are also functionally regulated through their interactions with various lipid molecules within the TEMs. As discussed above, the binding of cholesterol to the pockets formed by the transmembrane domains of tetraspanins regulates their conformations and functions. Tetraspanins also bind to glycosphingolipids (GSLs), which comprise membrane lipids formed by the attachment of glycans to a lipid core ceramide, and are classified into four main series according to their synthesis pathways: globo-series, ganglio-series, lacto-series, and the neolacto-series [49]. GSLs are synthesized within the Golgi apparatus and subsequently transported to the outer leaflet of the plasma membrane, where they regulate the structure and dynamics of the plasma membrane, including clustering, membrane curvature, and the internalization of bacterial toxins and glycosylated cargo glycoproteins [50,51,52,53]. B3GNT5 (UDP-GlcNAc:BetaGal beta-1,3-N-acetylglucosaminyltransferase 5), a key enzyme anchored in the Golgi membrane, contributes to the biogenesis of lacto-series glycosphingolipids. We recently found that SPPL3 (signal peptide peptidase like 3) controls the levels of lacto-series glycosphingolipids in the plasma membrane by cleaving and releasing B3GNT5 from the Golgi membrane for its subsequent degradation by the lysosome. SPPL3 deficiency resulted in the massive accumulation of lacto-series glycolipids, which affected the internalization of TSPAN8 and other tetraspanins from the plasma membrane [27]. Furthermore, super-resolution imaging revealed the colocalization of TSPAN8 proteins with the glycosphingolipid GM1 at the plasma membrane. Interestingly, the silencing of TSPAN8 expression reduced the presence of GM1 at the cell surface [54]. This study suggests that TSPAN8 and GM1 are mutually regulated in the plasma membrane.

Overall, TSPAN8 and other tetraspanin members dynamically interact with a wide array of molecules, including proteins and lipids within the plasma membrane. They coordinate the formation of TEMs that serve as critical signaling platforms for regulating diverse cellular behaviors, including cell migration/invasion, protein trafficking, and cell–cell communications during normal physiological functions as well as disease progression [55]. It is important to point out that the TSPAN8 protein is a small protein with four TM domains integrated into the membrane. Hence, it is unlikely that TSPAN8 can directly interact with many different protein partners with distinct functions. Instead, it is more likely that TSPAN8 and other tetraspanins act as organizers of TEMs by bringing together diverse proteins, lipids and other molecules through direct and indirect network interactions. Different techniques, including biochemical approaches with different detergents and high-resolution fluorescence methods, have been employed for the analysis of TEMs. It is important to note that the variations in methodologies in different studies are likely to introduce some biases. Moreover, the components of TEMs are tightly regulated, cell-context specific, and highly dynamic, even in the same cell. The interplay between TSPAN8 and its specific molecular partners within the TEMs in different contexts likely modulates its diverse and dynamic cellular functions.

## 6. Role of Plasma Membrane TSPAN8 in Cellular Signal Transduction

To date, no natural ligands and catalytic activities for TSPAN8 and other tetraspanins have been identified. However, TSPAN8 is known to form a complex with a diverse range of transmembrane receptors in various pathways and regulate their activities. In this section, we mainly discuss the role of TSPAN8 in the Wnt/β-catenin, EGFR, mTOR and hedgehog pathways (Figure 2).

TSPAN8 plays multifaced roles in the Wnt/β-catenin-signaling pathway. In the absence of Wnt signaling activation, β-catenin binds to E-cadherin in the plasma membrane. Upon Wnt signaling activation, β-catenin is translocated to the nucleus, where it functions as a transcriptional cofactor. A study on colorectal cells showed that TSPAN8 directly interacted with β-catenin and enhanced β-catenin expression, which then bound to the TSPAN8 promoter and promoted TSPAN8 transcription [56]. Similarly, TSPAN8 expression was reported to stabilize β-catenin, which in turn enhanced TSPAN8 transcription in melanoma cells [57]. Of note is that both studies did not address the specific cellular location of the interaction between β-catenin and TSPAN8. TSPAN8 might also regulate β-catenin transcription by binding to NOTCH2, a receptor of the Notch signaling pathway [58]. We recently showed that TSPAN8 interacts with LGR5 (leucine-rich repeat-containing G-protein coupled receptor 5), a G protein-coupled receptor involved in the Wnt signaling pathway, to maintain the quiescence of mammary stem cells [59].

The epidermal growth factor receptor (EGFR), which plays a crucial role in many key cellular processes such as cell proliferation, survival, motility, and differentiation [60], has been identified as a strong TSPAN8-binding partner [47]. Several studies have shown that EGF and TSPAN8 expressions are linked in a concentration- and time-dependent manner. For instance, TSPAN8 knockdown attenuated the effects of EGF on gastric cancer cell proliferation and invasion [61], while the suppression of endogenous EGF expression by KDM2A, a histone demethylase, decreased TSPAN8 expression in breast cancer cells [62]. Furthermore, SOX9 was identified as a key transcriptional regulator of TSPAN8 expression in response to EGF stimulation in pancreatic cancer cells. The EGF-SOX9-TSPAN8-signaling cascade was shown to regulate cancer cell invasion and metastasis [63]. Interestingly, CD9 and CD82 have also been found to form a complex with EGFR at the cell surface [64,65].

Additionally, TSPAN8 interacts with protein patched homolog 1 (PTCH1), a key transmembrane receptor of the sonic Hedgehog (SHH) signaling pathway. This interaction facilitates the recruitment of the deubiquitinating enzyme ATXN3 to inhibit the degradation of the SHH/PTCH1 complex. As a result, the protein SMO (smoothened) translocates to the cilia and triggers the expression of downstream target genes regulated by the hedgehog pathway [66].

Multiple independent studies investigating single nucleotide polymorphisms (SNPs) and copy number variants (CNVs) have unmasked an association between the genomic locus containing *LGR5*/*TSPAN8* and an increased risk of type 2 diabetes in different patient cohorts [67,68,69,70,71]. However, the significance of this association needs to be validated in additional studies. Notably, TSPAN8 knockout mouse models did not display apparent alterations in fasting insulin and glucose levels [70]. Nevertheless, TSPAN8 expression levels were found to be significantly reduced in the blood samples of diabetic nephropathy (DN) patients [72]. miR-543 was identified as a key regulator for TSPAN8 downregulation in kidney tissues of DN mice [73]. In an in vitro cell culture system, TSPAN8 was found to form a complex with Rictor (rapamycin-insensitive companion of mammalian target of rapamycin), a crucial component of the mammalian target of rapamycin complex 2 (mTORC2), to regulate high glucose-induced autophagy [72]. Interestingly, TSPAN8 also formed a complex with integrin α3 and Rictor in glioma cells [74]. This complex appeared to be necessary for the activation of mTORC2, as TSPAN8 knockdown prevented the assembly of mTOR-Rictor and the downstream phosphorylation of AKT and PKCα.

## 7. Involvement of TSPAN8 in the Biogenesis and Functions of Exosomes

Extracellular vesicles (EVs) are nanometer-sized structures enclosed by phospholipid bilayer membranes and released by most cells under both normal and pathological conditions [75]. They play a vital role in intercellular communication by delivering biological and genetic materials between cells [76]. Exosomes, a major subtype of EVs, typically range in size from 30 to 100 nm and are formed through the termination of the endocytic recycling pathway [77]. The process begins with the formation of early endosomes (EEs) at the trans-Golgi network or internalized plasma membrane microdomains. These EEs are then directed to multivesicular bodies (MVBs), where they incorporate cargo through the inward budding of intraluminal vesicles (ILVs), and then are secreted outside the cells as exosomes [78]. Exosomes can be isolated from various body fluids, including serum, saliva, and blood plasma. They are secreted by diverse cell types and partake in a wide array of physiological and pathological processes, depending on their cellular origins [79].

A notable characteristic of exosomes is their rich content of tetraspanins, with CD9, CD63, and CD81 being often regarded as pan molecular markers [80]. A comprehensive analysis using high-throughput quantitative proteomics revealed that approximately 45% of the exosome proteome is associated with the TEM interaction network [43]. The composition of lipids and proteins at the surface of exosomes determines the selectivity and specificity of target cells for precise cell-to-cell communication [79,81]. Tetraspanins cluster on exosome surfaces to interact with other proteins and organize themselves into TEMs [4,82]. Through these microdomains, exosomes can selectively target the membrane proteins of recipient cells [79,83]. For example, specific integrins, through their interactions with tetraspanins and other components of TEMs on the surface of exosomes derived from different cancers, determine the uptake of exosomes by organ-specific cells, a key step in the tissue-specific metastasis of primary cancer [84,85]. Notably, tetraspanins also appear to play a role in determining the composition of small RNA packaged into exosomes, although the underlying mechanism remains unclear [79]. Overall, tetraspanins contribute to various aspects of exosome biology, including biogenesis, cargo selection and the specificity of exosome uptake by recipient cells [86,87].

**Figure 3:**
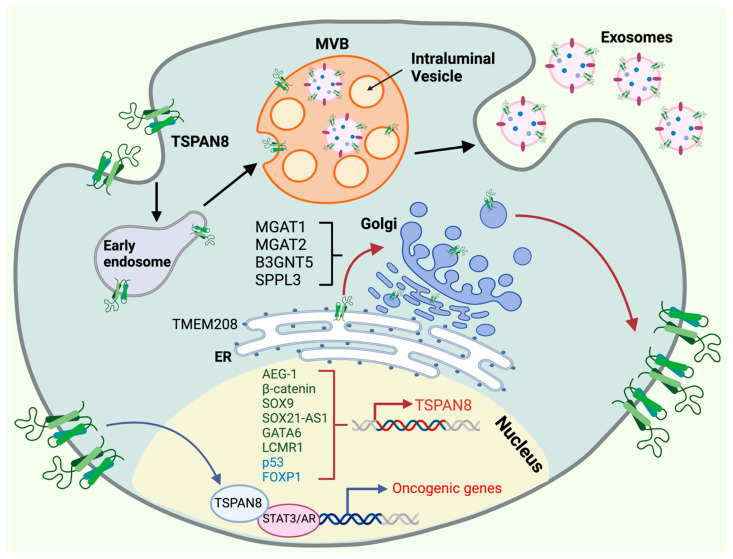
Molecular regulation of TSPAN8′s oncogenic functions. The oncogenic functions of TSPAN8 are controlled by multiple mechanisms, including transcriptional activation and repression, ER targeting, Golgi modification, cell-surface presentation, exosome biogenesis and nuclear translocation. TSPAN8 transcription is governed within the nucleus by a network of activators (dark green) and repressors (light blue). TSPAN8 protein is targeted through the ER by TMEM208, undergoing post-translation modifications at the Golgi apparatus before it moves to the cell surface. The presentation of TSPAN8 in the cell surface is regulated by a couple of proteins in the Golgi, including MGAT1/2, SPPL3 and B3GNT5. TSPAN8 can translocate back to the nucleus and serve as a transcriptional cofactor for the transcription factors, such as STAT3 and AR, to promote the expression of oncogenic genes. TSPAN8 also plays a pivotal role in exosome biogenesis, a process that is initiated by the formation of early endosomes (EEs) through vesicle uptake and recycling which subsequently progress into multivesicular bodies (MVBs), where cargo is incorporated into intraluminal vesicles (ILVs). Exosomes are ultimately formed and released from the cell. Created with BioRender.com, accessed on 16 January 2024.

TSPAN8 has been shown to be an important component of exosomes derived from diverse cancer cell types (Figure 3), where its expression is significantly elevated [88,89]. It appears to play a critical role in EV production, release, and attachment to target cells [90,91]. Moreover, TSPAN8-expressing exosomes are actively involved in tumor initiation, progression, and metastasis [85,87,88,89,90,91,92,93,94,95,96,97,98,99]. For example, one study reported that in gastric cancer, TSPAN8 expression in exosomes showed a positive effect on cell growth and invasion [94]. Another study reported that in pancreatic cancer cells with high TSPAN8 expression, tumor-derived EVs enhanced the maturation and activation of endothelial and fibroblast cells in the tumors [95]. TSPAN8 and CD44v6 mark pancreatic cancer stem cells (CSCs) and play a critical role in exosome biogenesis, loading, and delivery. The CSC-derived exosomes can reprogram neighboring non-CSCs and host cells and drive tumor progression through a series of activities, such as activating signaling cascades and promoting epithelial–mesenchymal transitions, transcriptions, translations, and miRNA processing in non-CSCs [98,99,100]. In rat adenocarcinoma models, TSPAN8 overexpression was shown to facilitate the selective recruitment of CD106 and CD49d to promote exosome–endothelial cell binding and internalization. This led to the vascular endothelial growth factor (VEGF)-independent regulation of angiogenesis and vascular remodeling [87]. Additionally, TSPAN8-competent exosomes derived from tumor cells were also shown to promote matrix degradation and shape the tumor microenvironment, which in turn favored tumor growth and metastasis [101]. Studies involving the transplantation of TSPAN8 and CD151 single- and double-knockout cancer cells into wild-type or autochthonous and syngeneic recipient mice have further unraveled the complex roles of these tetraspanins in host- versus tumor-derived exosomes [92]. Circulating EVs overexpressing TSPAN8 have been explored as a non-invasive biomarker for various cancer types and will be discussed in the last section of this review.

Recently, multiple members of tetraspanins have also been identified in novel cellular organelles known as migrasomes, which are large EVs closely associated with cell migration [102]. In addition to migration, migrasomes have important roles in various other physiological processes, including mitochondrial quality, organ morphogenesis, intercellular communication, and mRNA/protein transfer between cells [103,104,105,106]. These migrasomes were initially observed as pomegranate-like vesicular structures approximately ranging in size from 500 to 3000 nm and containing numerous small intraluminal vesicles within them. Migrasomes form on retraction fibers at the trailing edge of migrating cells and are released as cells move away [102,107]. Similar to exosomes, migrasomes also show high expression of tetraspanin proteins, with TSPAN4 as a key marker [108]. However, the specific roles of TSPAN8 in migrasome biogenesis and functions remain unexplored.

## 8. TSPAN8 Promotes Cell Migration/Invasion and Cancer Metastasis

Cell migration is fundamental to diverse biological processes, such as tissue formation, hemostasis and regeneration, that occur under physiological conditions as well as cancer cell invasion and metastasis occurring under pathological conditions [109,110]. A study using a TSPAN8 knockout mouse model demonstrated its role in enhancing cell motility, particularly during skin wound healing [111]. TSPAN8 is prominently expressed in melanoma, a particularly aggressive form of skin cancer with a high metastatic potential, where it is involved in regulating the invasive properties of these malignant cells [112,113,114]. TSPAN8 has also been identified as an important modulator of motility in colorectal carcinoma cells [47]. Similarly, TSPAN8 overexpression in gastric cancer and nasopharyngeal carcinoma cell lines has been shown to promote cell invasion [115,116,117]. Elevated TSPAN8 expression has been linked to enhanced invasions of hepatocellular carcinoma (HCC) cells in vitro and increased metastasis in HCC animal models [118,119]. In one study, exogenous expression of TSPAN8 promoted the migration/invasion of low-invasive esophageal carcinoma cell lines in vitro and their ability to invade surrounding tissues and develop lung metastasis in vivo [120]. Astrocyte-elevated gene-1 (AEG-1) is known as an important oncogene that is overexpressed in many cancers and promotes tumor progression and metastasis [121]. Interestingly, in one study, AEG-1 promoted the upregulation of *TSPAN8,* and knocking down *TSPAN8* significantly reduced the migration and invasion of AEG-1-overexpressing HCC cells [122]. TSPAN8 expression in cancer cells was also found to promote epithelial-mesenchymal transition (EMT), a key process for cells to acquire the potential to migrate and invade surrounding tissues [123]. Interestingly, various subtypes of cancer-associated fibroblasts (CAFs)—an important stromal cell population in the tumor microenvironment that assists in EMT, the invasion of cancer cells and cancer metastasis—are characterized by high TSPAN8 expression in high-grade serous ovarian cancer (HGSOC). However, whether enhanced TSPAN8 expression in CAFs and other stromal cells has an effect on the regulation of EMT, the invasion of cancer cells and cancer metastasis remains largely unknown [124].

Cell migration and invasion are regulated by integrins, cadherins, small GTPases such as Rac and Rho/ROCK signaling, and extracellular matrix (ECM) proteases such as matrix metalloproteinases (MMPs) [125]. Integrins and cadherins are the two main classes of transmembrane cell adhesion receptors. Cadherins (calcium-dependent adhesions) are important for forming junctions that facilitate cell–cell adhesion. Integrins, on the other hand, mainly mediate adhesion between a cell and its surrounding ECM. Unlike cadherins, all integrins are heterodimeric molecules containing an α and β subunit, held together through non-covalent interactions. Both subunits are proteins with a single transmembrane domain and a cytoplasmic c-terminus. The combination of specific α and β subunits determines the ligand specificity of integrins [126]. Tetraspanins, including TSPAN8, can form complexes with specific integrin heterodimers on the surface of cancer cells and enhance the binding of cancer cells to ECM components, providing stronger adhesion points for their movement and invasion into surrounding tissues (Figure 2B) [127]. TSPAN8 was found to colocalize and coimmunoprecipitate with an α6β4 integrin in human pancreatic adenocarcinoma, with the activation of protein kinase C (PKC) likely promoting this interaction [128]. Interestingly, the expressions of α6β4 and TSPAN8 were primarily restricted to tumor cells, while other integrins and tetraspanins were present in the surrounding stromal cells. TSPAN8 is also abundant in tumor-derived exosomes and is able to associate with integrins, particularly α4 and β4 integrin chains, in the target cells [83]. It is important to note that the integrin heterodimers and downstream signaling pathways regulated by TSPAN8 are dependent on the cellular context and the type of cancer. Further research is needed to deepen the understanding of the mechanisms and consequences of TSPAN8–integrin interactions. E-cadherin, together with its adapter p120-catenin, is known to play a critical role in cell adhesion and the maintenance of tissue integrity [129]. A protein complex containing E-cadherin, p120-catenin and TSPAN8 has been identified to play an important role in cell motility and cancer metastasis in colon carcinoma and breast cancer [90,130]. While integrins lack intrinsic catalytic activity, they can form focal adhesions (specialized structures that link the cell’s cytoskeleton to the ECM) and initiate FAK (focal adhesion kinase)-mediated signaling cascades [131]. TSPAN8–integrin interactions promote focal adhesion formation and activate downstream signaling pathways that regulate cytoskeletal rearrangements and cancer cell motility [132]. TSPAN8 is also important for FAK activation, forming a complex with activated FAK in primary malignant glioma tissues and glioma cell lines [133].

A disintegrin and metalloprotease (ADAM) and matrix metalloproteinases (MMPs) are structurally related, zinc-dependent metalloproteinases that are responsible for degrading various components of the ECM [134]. TSPAN8 has been implicated in regulating the activity of multiple ADAMs and MMPs, particularly during cancer invasion and metastasis (Figure 2C). Several assays have demonstrated the physical interaction between ADAM17 and TSPAN8 in the plasma membrane, which enhances the ADAM17-mediated release of TNF-α (tumor necrosis factor α) and thus leads to increased tissue inflammation and tumorigenesis [135]. Interestingly, TSPAN8 has been found to contribute to the migration, invasion, and metastasis of esophageal carcinoma and HCC cells by increasing the expression of a specific isoform (ADAM12m) of ADAM12, which is implicated in the metastasis of various epithelial cancers [118,120]. In a skin reconstruction model mimicking melanoma dermal penetration through the dermal–epidermal junction, an early step in melanoma dissemination, TSPAN8-positive melanoma cells were shown to cooperate with adjacent keratinocytes in the epidermis to promote the activation of proMMP-9, an enzyme involved in skin ECM degradation [113]. A monoclonal TSPAN8 antibody was able to block the activation of proMMP-9 and subsequent dermal invasions by melanoma cells. However, in vivo studies in mouse cancer models suggest that TSPAN8 enhances cancer cell motility and migration mainly by recruiting integrins, whereas CD151 efficiently recruits and activates MMP9 and MMP13 to facilitate the degradation of the ECM and the invasion of tumor cells [92,93,101,136]. In addition to cancers, TSPAN8 may play a role in other diseases by regulating the functions of a few other metalloproteases. For example, TSPAN8 binds to and recruits meprin β, a metalloprotease involved in ectodomain shedding activity at cell surfaces, to TEMs in the plasma membrane. Meprin β plays a role in cleaving the amyloid precursor protein, which leads to the release of neurotoxic amyloid β peptides that are implicated in Alzheimer’s disease [137,138]. Furthermore, TSPAN8 physically interacts with ECE1 (endothelin converting enzyme 1), a membrane-bound metalloprotease that catalyzes the proteolytic activation of big endothelin-1 and regulates its activity [139]. ACE2 (Angiotensin-converting enzyme 2) is the primary receptor in host cells that enables SARS-CoV-2 attachment and cell entry through interactions with the viral spike protein. CD151, CD9, and TSPAN8 have been shown to facilitate the entry of coronaviruses into host cells by recruiting host cell receptors, including ACE2, and proteases into TEMs [140,141]. However, a recent study showed that TSPAN8 facilitates SARS-CoV-2 infection through a mechanism independent of the ACE2-spike protein interaction [142].

## 9. TSPAN8 as a Genetic Marker and Key Regulator of Normal Tissue Stem Cells and Cancer Stem Cells

The expression of *TSPAN8* mRNA is restricted to defined cell lineages, and TSPAN8 has emerged as a key marker and regulator of stem/progenitor cells in different tissues. We previously identified TSPAN8 and LGR5 as molecular markers of quiescent mammary stem cells [143] and unraveled that TSPAN8 physically interacts with LGR5 to form a functional complex in these cells to regulate their quiescent status (Figure 2D) [59,144]. Interestingly, TSPAN8 expression also defines the progenitor subset in the luminal population of mouse mammary glands [143]. A recent study reported that *BRCA2* mutation-associated breast cancers potentially originate from TSPAN8^+^ luminal progenitor cells [145]. The suppression of *TSPAN8* expression by the transcriptional factor FOXP1 is essential for quiescent mammary stem cells to re-enter cell cycle for ductal morphogenesis [59,144]. A knockout of *FOXP1* led to the constitutive expression of TSPAN8 and prevented the exit of mammary stem cells from quiescence, profoundly blocking the mammary gland development in mice. TSPAN8 has also been identified as a specific marker for spermatogonia stem cells [146]. In rats, TSPAN8 down-regulation in Sertoli cells plays an obligatory role in the division and differentiation of male germ cells into sperms during puberty. Remarkably, sperm production, in one study, was almost completely blocked in a transgenic rat model where TSPAN8 downregulation in Sertoli cells was prevented from puberty up to adulthood [147].

**Figure 4:**
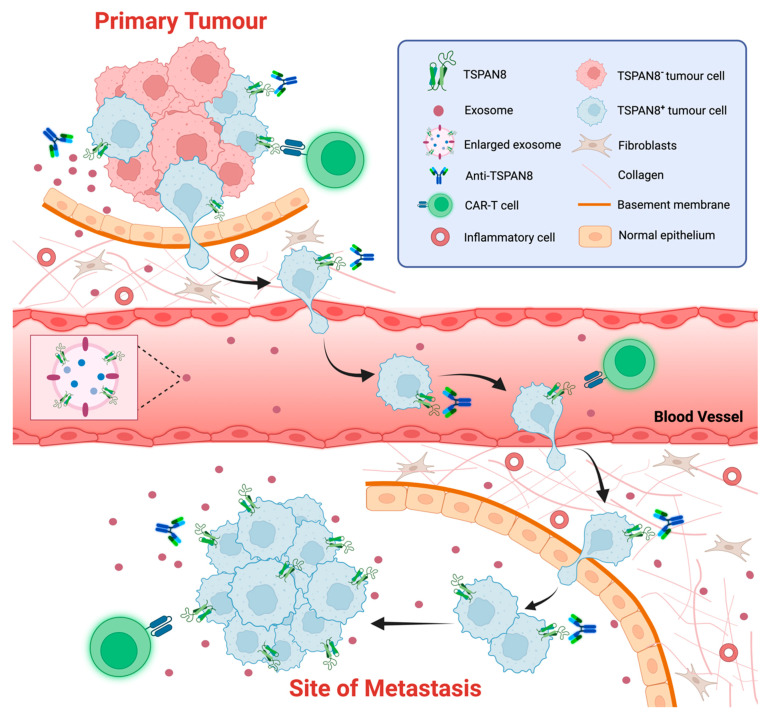
TSPAN8 as a biomarker and therapeutic target of cancer. The schematic representation of strategies targeting TSPAN8 at different stages of tumor progression. TSAPN8 expression has been shown to be a molecular marker for cancer stem cells (CSCs) or cancer-initiating cells (CICs). Multiple monoclonal anti-TSPAN8 monoclonal antibodies have been shown to impair cancer progression. They may restrain primary tumor growth and block the migration/invasion of cancer cells, which in turn prevents metastasis. Anti-TSPAN8 antibodies may also suppress the formation of tumor-promoting microenvironments by inhibiting the biogenesis and release of cancer cell-derived exosomes. Additionally, CAR-T cells targeting TSPAN8 may be a novel promising strategy for treatment of various epithelial cancers with TSPAN8 expression. Moreover, detection of TSPAN8-expressing circulating tumor cells or exosomes potentially represents a non-invasive approach for diagnosis and prognosis of various epithelial cancers. Created with BioRender.com, accessed on 16 January 2024.

TSPAN8 expression also marks cancer stem cells (CSCs) or cancer-initiating cells (CICs) in various cancer types (Figure 4). For instance, TSPAN8 is one of the markers used to characterize pancreatic cancer stem cells (Pa-CSCs) [100]. In another study, TSPAN8, alongside CD44v6, an α6β4 integrin, and CD133, were found to be upregulated in pancreatic cancer-initiating cells (Pa-CIC), where they conferred in vivo growth and metastatic advantages [148]. TSPAN8 expression was elevated in breast CSCs, where high levels of TSPAN8 conferred on CSCs resistance to chemotherapy [66]. Mechanistically, TSPAN8 was shown to promote cancer cell stemness in breast cancer through the activation of the sonic Hedgehog (SHH) signaling pathway, which led to the upregulation of stemness-related genes [66]. Similarly, a positive regulatory loop between β-catenin and TSPAN8 regulated the expression of stemness genes for the maintenance of cancer stemness and the sphere-forming ability of colorectal cancer cells [56].

## 10. Regulation of *TSPAN8* Transcription

Given that TSPAN8 is upregulated and partakes in the progression of various cancers, it is crucial to understand the molecular regulation of *TSPAN8* transcription. AEG-1 (astrocyte elevated gene-1), which promotes tumor progression and metastasis, upregulates *TSPAN8* transcription through the activation of the MEK/ERK signaling pathway [122]. β-catenin directly promotes *TSPAN8* transcription through a positive feedback loop [56,57]. The long non-coding RNA (lncRNA) SOX21 antisense RNA1 (SOX21-AS1), which is overexpressed in several cancers including HCC, colorectal cancers, and lung adenocarcinoma [149,150,151], acts as a positive co-regulator to promote *TSPAN8* transcription by the transcription factor GATA6, therefore increasing the migration and invasion of cancer cells [152]. In response to EGF stimulation, the transcription factor SOX9 activates *TSPAN8* expression and promotes cell invasion [63]. In a study using large scale RNAi screens, several genes, including GSK3β, PTEN, IQGAP1, TPT1 and LCMR1, were identified to inhibit or enhance *TSPAN8* transcription. In particular, LCMR1 (lung cancer metastasis-related protein 1) enhanced *TSPAN8* expression and promoted the invasion of melanoma cells [112]. Alternately, in non-invasive melanoma cells, the transcription factor p53 binds to consensus-binding sites in the promotor region of *TSPAN8* and directly suppresses *TSPAN8* transcription [153]. Moreover, we have previously uncovered that FOXP1 suppresses *TSPAN8* transcription in the basal cell population of mouse mammary epithelium, which is critical for the exit of mammary stem cells from quiescence [59].

## 11. Novel Functions of TSPAN8 in the Nucleus

As described above, tetraspanins are transmembrane proteins that mainly localize and function in the plasma membrane. Studies have shown that TSPAN8 can release from the plasma membrane and translocate to the nucleus in multiple cancer cells (Figure 3). This process seems to be assisted by TSPAN8 palmitoylation and cholesterol binding [154]. Moreover, the phosphorylation of TSPAN8 by AKT enables the binding of TSPAN8 to 14-3-3θ and importin ß1, which is essential for its nuclear translocation in response to the activation of EGF-EGFR signaling. Nuclear TSPAN8 interacts with the transcription factor STAT3 to activate the transcription of cancer-promoting genes including MYC, BCL-2 and MMP9, which in turn promotes tumorigenesis [39,155]. Interestingly, a new humanized monoclonal antibody targeting the LEL domain of TSPAN8 was able to suppress the release of TSPAN8 from the plasma membrane and its subsequent nuclear translocation. Furthermore, androgen was also found to induce a nuclear localization of TSPAN8, resulting in the formation of TSPAN8 and an androgen receptor (AR) complex in prostate cancer cells [156]. Nuclear TSPAN8 is required for the transcriptional functions of AR and AR variant 7 (AR-V7) [157]. Of note is that the detailed molecular process for the translocation of a plasma membrane-integrated protein to the nucleus remains unclear. However, the accumulating evidence suggests that distinct plasma membrane proteins, including cell signaling receptors and cell adhesion proteins, are able to move to the nucleus to execute a non-canonical function under certain circumstances [158,159].

## 12. TSPAN8 as a Biomarker and Therapeutic Target of Cancer

TSPAN8 was originally identified as a human tumor-associated antigen found to be overexpressed in many different epithelial cancers, including lung, liver, gastric, esophageal, colorectal, and ovarian carcinomas [130,160,161]. Accumulating evidence suggests that TSPAN8 plays an oncogenic role in the initiation, progression, and metastasis of different epithelial cancers. In line with this, there is a positive correlation between high TSPAN8 expression and clinicopathological characteristics of an aggressive tumor, including tumor differentiation, invasion depth, lymph node metastasis, and clinical stage [162,163,164,165,166]. However, there is also some evidence against this [167,168].

The potential of employing TSPAN8 mRNA and proteins as novel blood biomarkers for different epithelial cancers has been explored. Based on a systematic large-scale meta-analysis on the blood samples, a panel of mRNAs of four genes—TSPAN8, LGALS4, COL1A2, and CEACAM6—were identified as putative markers of colorectal cancer [169,170,171]. Genome-wide microarray analysis identified *TSPAN8* as a gene that is significantly upregulated in gastric cancers compared to normal gastric mucosae, suggesting its use as a novel molecular marker for the detection of circulating gastric cancer cells in the peripheral blood [172]. Another study using proteomic analysis showed the selective enrichment of the TSPAN8 protein in EVs from metastatic NSCLC (non-small cell lung cancer) cell lines. Consistent with this, serum EVs from patients with stage III premetastatic NSCLC tumors displayed high TSPAN8 levels. This study highlights the potential of testing TSPAN8 protein levels in EVs from blood for prognosing the metastasis of NSCLC patients [96]. Collectively, TSPAN8 may represent a promising candidate for use as blood-based biomarkers for cancer screening.

Therapeutic monoclonal antibodies (mAbs) targeting different tetraspanin members including TSPAN8, CD9, CD37 and CD151 have been explored in preclinical models and clinical trials for the treatment of hematological malignancies and carcinomas [173,174,175,176,177]. Several monoclonal antibodies against TSPAN8 have been developed and showed a significant inhibition of tumor growth and metastasis in preclinical cancer models. For example, the monoclonal antibody Ts29.2 that specifically targets human TSPAN8 showed significant efficacy in pre-clinical models of CRC [178]. Similarly, angiogenesis induced by TSPAN8 in rat tumor models could be effectively inhibited by the anti-rat TSPAN8 specific antibody D61.A [179]. Interestingly, the antibodies were highly selective in inhibiting sprouting endothelial cells and were effective regardless of whether the tumor cells themselves expressed TSPAN8 or not. In recent years, several monoclonal TSPAN8 antibodies specifically targeting the LEL of human TSPAN8 have been developed and tested for their potential in treating different solid tumors. For instance, the humanized monoclonal antibody hT8Ab4 showed an anti-tumor effect in multiple cancers associated with the nuclear translocation of TSPAN8 [39]. Another humanized monoclonal antibody, C4 scFv-Fc, displayed high affinity binding, even at sub-nanomolar concentrations, to amino acids 140-205 within TSPAN8 LEL in a conformation-dependent manner [180]. This antibody also significantly reduced the invasion of metastatic colorectal cancer (mCRC) cell lines that express TSPAN8 compared to non-mCRC cell lines. Furthermore, the monoclonal antibody TSPAN8–LEL IgG was shown to significantly reduce the incidence of epithelial ovarian cancer metastasis in vivo without causing severe toxicity [177]. The exact mechanisms for the tumor-inhibitory effects of these antibodies are yet to be elucidated. The binding of these antibodies to cell-surface TSPAN8 may lead to alternations in TSPAN8-enriched TEMs and subsequent cell signaling cascades.

The cell-surface expression of TSPAN8 can also be used to target and destroy cancer cells, such as through antibody–drug conjugates or CAR-T (chimeric antigen receptor T-cells). In mouse CRC xenograft models, the radiolabeled antibody Ts29.2 demonstrated high specificity in localizing to tumors as well as high efficacy—up to 70%—in reducing tumor burdens [181]. CAR-T therapy is a ground-breaking immunotherapy for certain types of cancer. However, the identification of suitable tumor-specific antigens for this therapy remains challenging. To discover effective target candidates for pancreatic ductal adenocarcinoma, a strategy combining flow cytometry screenings, bioinformatic expression analyses, and a cyclic immunofluorescence platform was developed. CLA, CD66c, CD318 and TSPAN8 were identified as putative target candidates from 371 tumor antigens. Remarkably, CAR-T cells specifically targeting TSPAN8 showed high efficacy, with complete tumor eradication in some cases. These findings highlight TSPAN8 as a promising candidate, with high potential for successful clinical translation [182]. Another in vitro study aimed at developing a novel spacer for CAR-T cells to improve their functionality against membrane-proximal epitopes demonstrated efficient engagement of TSPAN8-specific CAR-T cells. Remarkably, the transplantation of these cells in vivo showed excellent tumor-killing efficacy [183].

## 13. Conclusions

The tetraspanin proteins have emerged as a new superfamily of integral membrane proteins that have similar plasma membrane topology and conserved molecular features. The members of the superfamily, including TSPAN8, have now been recognized as key organizers of the plasma membrane. However, a more in-depth understanding is required on how tetraspanins recruit different molecules, including proteins and lipids, to facilitate the formation of TEMs, which are dynamic and specialized platforms mediating diverse biological processes. Accumulating evidence clearly reveals that TSPAN8 is predominantly localized in the plasma membrane of cells. Cell-surface TSPAN8 plays an important role in diverse molecular and cellular events, including cell adhesion, migration, invasion, signaling transduction and exosome biogenesis, through direct or indirect interactions with a wide range of protein and lipid partners in TEMs. While different tetraspanin members are believed to be functionally redundant, TSPAN8 has been associated with some unique and indispensable functions in many cellular contexts. However, several gaps remain in the comprehensive understanding of TSPAN8 biology. Recently, a cryo-EM structure of a complex formed by TSPAN15 and its binding partner ADAM10 has been resolved [184]. The structure of TSPAN8 within the plasma membrane and complexes of TSPAN8 and its binding partners has not been resolved to date, resulting in insufficient insights into its functional specificity and redundancy. While it is known that the cell-surface presentation and functions of TSPAN8 and other tetraspanins are governed by ER targeting, modifications at the Golgi apparatus and other trafficking processes, the detailed molecular mechanisms underlying these processes remain largely unknown. Furthermore, recent evidence has shown that TSPAN8 translocates from the plasma membrane to the nucleus, where it executes novel functions as a cofactor of certain transcriptional factors, such as STAT3 and androgen receptors, for their transcriptional activity. However, the mechanisms by which TSPAN8 is extracted from the plasma membrane and how membrane-free TSPAN8 maintains stable protein folding for its novel functions are unclear. The transcription of *TSPAN8* is tightly controlled, leading to tissue- and cell-specific expression patterns for *TSPAN8* mRNA under normal physiological conditions. Interestingly, *TSPAN8* mRNA is highly expressed in various epithelial cancers and can serve as a genetic marker for cancer stem cells or cancer-initiating cells in some cases. Furthermore, several transcriptional factors have been shown to bind to the *TSPAN8* promoter and positively or negatively regulate its transcription. Since most of these studies are based on in vitro culture systems, further in vivo studies are needed to address how *TSPAN8* transcription is switched on and its effects and significance in promoting tumor initiation, progression, and metastasis in different epithelial cancers. Tumor-derived exosomes play a critical role in shaping the niche for the growth of primary tumors and their metastasis. While accumulating evidence suggests that TSPAN8 plays a role in the biogenesis and functions of tumor-derived exosomes, the underlying mechanisms by which TSPAN8 regulates communications between cancer cells and their microenvironment of various cancer types need to be explored further. Numerous studies have implied the potential of detecting TSPAN8 expression in circulating tumor cells or exosomes as a blood biomarker for cancer diagnosis, prognosis, and metastasis prediction. Apart from their diagnostic applications, TSPAN8 has also been recognized as a promising target for several therapeutic strategies, as multiple monoclonal anti-TSPAN8 antibodies have shown significant efficacy in pre-clinical cancer models, and CAR-T cells have efficiently targeted TSPAN8-expressing cancer cells (Figure 4). These studies have paved the way for using TSPAN8 as a non-invasive biomarker for cancer diagnosis and prognosis and as a therapeutic target for the treatment of various epithelial cancers. However, further evaluation and validation in clinical settings are needed in the future.

## Data Availability

Data reported in this paper will be shared by the lead contact upon request. This paper does not report an original code. Any additional information required to re-analyze the data reported in this paper is available from the lead contact upon request.
